# Superior Semicircular Canal Dehiscence in a Patient with Ehlers-Danlos Syndrome: A Case Report

**DOI:** 10.7759/cureus.1141

**Published:** 2017-04-06

**Authors:** Lawrance K Chung, Carlito Lagman, Daniel T Nagasawa, Quinton Gopen, Isaac Yang

**Affiliations:** 1 Neurosurgery, David Geffen School of Medicine, University of California, Los Angeles; 2 Department of Head and Neck Surgery, David Geffen School of Medicine, University of California, Los Angeles

**Keywords:** superior semicircular canal dehiscence, ehlers-danlos syndrome

## Abstract

Superior semicircular canal dehiscence (SSCD) is a bony defect in the middle cranial fossa floor that results in an abnormal connection between the inner ear and cranial vault. Although the etiology of SSCD remains unclear, an inappropriately thin or fragile temporal bone likely predisposes an individual towards developing SSCD. Ehlers-Danlos syndrome (EDS) constitutes a group of genetic connective tissue disorders caused by a defect in the production, processing, or structure of collagen, or its associated proteins. The possible association between SSCD and EDS has not been previously described in the literature. We herein report a case of a 50-year-old female with EDS-hypermobility type who presented with a 15-year history of migraines, vertigo, and tinnitus. The patient was subsequently diagnosed with bilateral SSCD and underwent a right middle fossa (pre-auricular infratemporal) craniotomy for SSCD repair. She reported significant improvement in her auditory and vestibular symptoms, with the exception of continued mild dizziness and disequilibrium at the 3-month follow-up. Due to the rare reports of auditory symptoms in EDS, this case study highlights the importance of considering an otological consultation for auditory manifestations in a patient with EDS and illustrates a potential association between EDS and SSCD.

## Introduction

Superior semicircular canal dehiscence (SSCD) describes a bony defect in the middle fossa floor directly above the superior semicircular canal. The defect creates a third window in the inner ear, in addition to the physiological round and oval windows. The abnormal connection between the middle cranial fossa and inner ear alters fluid flow in the cochlea and changes the air and bone conduction thresholds, resulting in a constellation of auditory and vestibular symptoms [1].

Ehlers-Danlos syndrome (EDS) constitutes a group of genetic connective tissue disorders caused by a defect in the production, processing, or structure of collagen, or its associated proteins. Ehlers-Danlos syndrome-hypermobility type (EDS-HT) is considered the most common and least severe of the EDS subtypes [2]. Affected individuals have hypermobile joints with arthralgia, myalgia, arthritis, and joint instability. Patients may also present with a number of cardiovascular, gastrointestinal, hematologic, ocular, gynecologic/obstetric, urologic, and neurologic symptoms. We herein present a case of SSCD in a patient with EDS-HT. To our knowledge, this is the first reported case of SSCD in a patient with EDS.

## Case presentation

### History and physical exam

A 50-year-old female with a history of EDS-HT presented to the clinic with a 15-year history of migraines, vertigo, tinnitus, and hyperacusis. She complained of amplification of chewing noises but denied hearing any other internal sounds. She reported that her vertigo was induced by movement and pressure changes, which trigger episodes of migraines and tachycardia. Her physical exam was unremarkable, with no evidence of nystagmus or cranial nerve deficits. On audiometry, she had a speech reception threshold of 10 dB and word recognition score of 100% bilaterally. She underwent further evaluation with high-resolution computed tomography (CT) imaging. Verbal consent to publish this case report was obtained from the patient and the study was approved by the institutional review board (IRB #13-001820).

### Imaging

**Figure 1:**
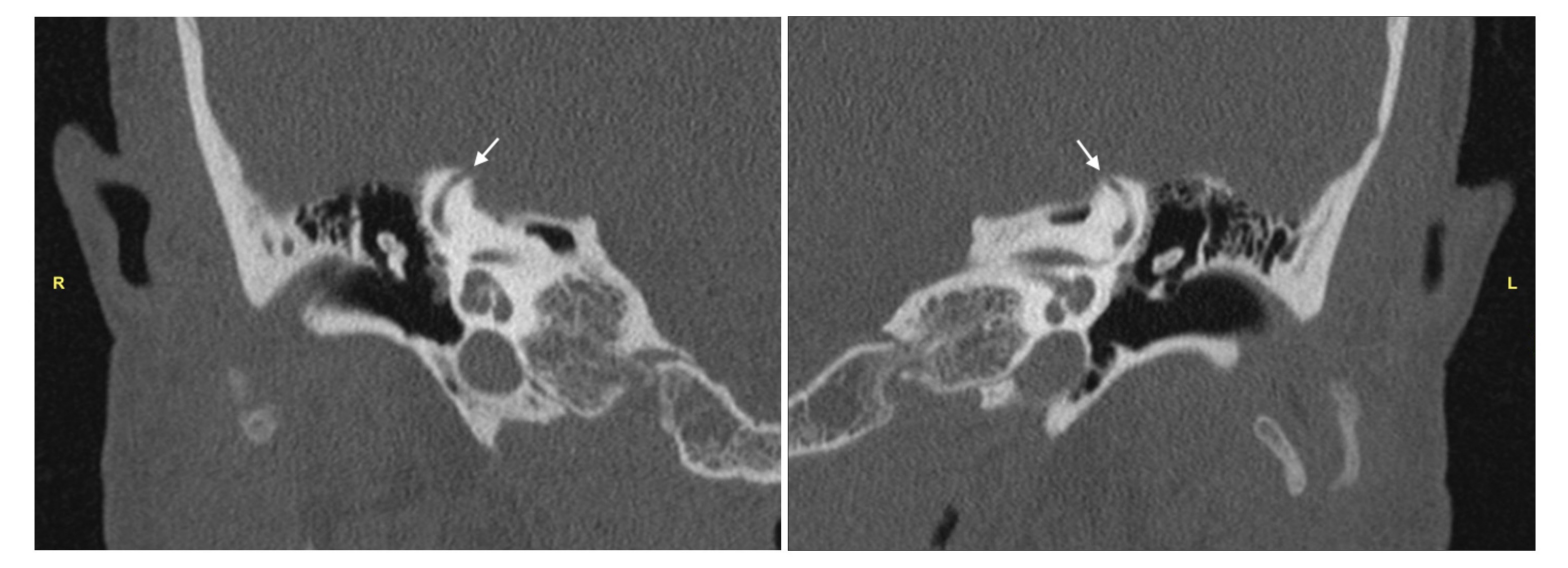
Computed Tomography of Temporal Bones High-resolution computed tomography scans of the temporal bones demonstrating the area of dehiscence (arrow) at the apex of the (R) right superior semicircular canal and (L) left superior semicircular canal

A high-resolution CT scan of the temporal bones demonstrated 5 mm (right) and 4 mm (left) dehiscent segments at the superior-most aspects of the superior semicircular canals, consistent with SSCD (Figure 1). Her constellation of auditory and vestibular symptoms was attributed to the presence of canal dehiscence.

### Management

At our institution, patients with bilateral SSCD undergo sequential repair, starting on the side of the larger defect. This allows for symptom improvement prior to evaluating the need for further repair on the contralateral side. Thus, our patient underwent a right minimally invasive middle fossa (pre-auricular infratemporal) approach for SSCD repair. A 4 cm curvilinear skin incision was made above the ear. A circular craniotomy 1.7 cm in diameter was then placed above the external auditory canal at the level of the zygoma. The arcuate eminence was identified using standard microdissection techniques and neuronavigation. The right semicircular canal dehiscence was identified along with numerous other bony defects on the middle fossa floor. Bone wax and autologous bone chips were used to repair the defects, and the craniotomy and incision were closed in standard fashion. The patient had a postoperative course complicated by nausea that was managed by a variety of anti-emetics. She was then discharged home in stable condition.

### Follow-up

The patient had an unremarkable follow-up period. At her three-month visit, she reported significant improvement in her auditory and vestibular symptoms, with the exception of continued mild dizziness and disequilibrium since the initial postoperative period. Of note, her tinnitus has completely resolved and her hearing has remained stable.

## Discussion

The constellation of symptoms between different syndromes may overlap and potentially cause difficulty in accurately diagnosing a patient. This is particularly true for diseases which have clinical manifestations in a number of different organ systems. Patients with SSCD are commonly affected by vertigo, hearing loss and to a lesser extent, headaches [3]. Our patient’s chief complaints of migraines and vertigo are also frequently described in patients with EDS-HT. One study reported that 78% of patients with EDS-HT experienced dysautonomia, including headaches and dizziness [4]. Furthermore, 50% of adults with EDS-HT have chronic, recurrent headaches [5].

Conversely, otological manifestations of EDS-HT are relatively uncommon. In 21 patients with EDS-HT, Castori, et al. found that 23.8% of the patients subjectively reported mild hearing impairment [6]. However, only three patients with EDS have been reported in the literature to develop significant hearing loss [7-9]. In two patients, their conductive hearing loss was attributed to otosclerosis, while the third was attributed to retraction of the tympanic membrane. Due to the rarity of hearing loss observed in patients with EDS, some authors have suggested that hearing loss in these patients may not actually have been a manifestation of EDS. This exact scenario appears to be the case in our patient, whose auditory symptoms were likely a result of bilateral SSCD, rather than EDS-HT. Thus, whether hearing loss remains a manifestation of EDS, or due to another etiology, remains unclear.

The underlying pathogenesis of SSCD is due to the formation of an abnormal fistula between the superior semicircular canal and middle cranial fossa. Although the exact cause of SSCD remains unclear, an inappropriately thin or fragile temporal bone likely predisposes an individual towards developing SSCD. Given that patients with EDS often manifest with a variety of musculoskeletal deficits due to aberrant collagen production, this association brings into question whether patients with EDS have a higher risk of developing SSCD.

While altered bone mass is not considered a cardinal feature of EDS, Dolan, et al. found that patients with EDS have both decreased bone mineral density and an increased risk of bone fractures compared to age-matched control patients [10]. Type 1 collagen constitutes the main organic component in bones; therefore, a patient with EDS may have a more delicate middle fossa floor that predisposes them to developing SSCD in the setting of a congenitally thin temporal bone and a secondary event (e.g., trauma, intracranial hypertension, temporal lobe erosion). However, due to the rarity of SSCD, large population studies will likely be required to uncover the true incidence and relationship between patients with EDS and SSCD.

## Conclusions

The clinical symptoms of both EDS-HT and SSCD may include headaches and vertigo. However, auditory findings in EDS are considerably less common and should prompt evaluation for another etiology, possibly through otological consultation. However, it is postulated that the pathophysiology of EDS potentially predisposes patients to a higher incidence of developing SSCD.
